# Identification of Causal Agents of Rust of *Saccharum* spp. and Assessment of Resistance to Brown Rust in *Erianthus arundinaceus* Clones and Their Offspring

**DOI:** 10.3390/plants14081221

**Published:** 2025-04-16

**Authors:** Jun-Lv Chen, Sheng-Ren Sun, Zhu-Qing Wang, Hua-Ying Fu, Huan-Yin Xu, Hai-Long Chang, San-Ji Gao, Qin-Nan Wang

**Affiliations:** 1 Institute of Nanfan & Seed Industry, Guangdong Academy of Sciences, Guangzhou 510000, China; junlvchen@163.com (J.-L.C.);; 2 National Key Laboratory for Tropical Crop Breeding, Haikou 571101, China; 3 National Engineering Research Center for Sugarcane, Fujian Agriculture and Forestry University, Fuzhou 350002, China; mddzyfhy@163.com

**Keywords:** sugarcane rust, *Bru1* gene, *Saccharum* spp. hybrids, *Erianthus arundinaceus*, germplasm resources

## Abstract

Sugarcane rust diseases are caused by *Puccinia melanocephala* (brown rust) and *Puccinia kuehnii* (orange rust), and significantly threaten the sustainable and stable development of the global sugarcane industry. *Erianthus arundinaceus* within the *Saccharum* complex is a potential germplasm resource for sugarcane breeding and is characterized by its tolerance of infertile land, drought, and diseases. However, the research on resistance to rust in *E. arundinaceus* clones and their offspring (F1 and backcross with modern sugarcane varieties) is limited. In this study, a total of 201 leaf samples from *Saccharum* spp. hybrids with rust symptoms were collected and screened for disease occurrence. PCR detection revealed that 17.9% and 34.8% of the samples were infected by *P. melanocephala* and *P. kuehnii*, respectively. Additionally, 12.9% of the samples were infected by both pathogens. A total of 88 clones of *E. arundinaceus* offspring and the parents plus 3 additional *E. arundinaceus* were selected for the identification of brown rust resistance by an artificial inoculation method. Among them, 61 clones displayed high resistance to brown rust. Molecular detection showed that 13 offspring of *E. arundinaceus* and 6 backcross parents of the “ROC” series exhibited the major resistance gene (*Bru1*) for brown rust. Unexpectedly, the *Bru1* gene was absent in 42 clones that were resistant to brown rust, suggesting that other resistance genes for brown rust likely exist in *E. arundinaceus* and their offspring. Our results offer some significant genetic resources for developing sugarcane cultivars with resistance against rust.

## 1. Introduction

Sugarcane (*Saccharum* spp. hybrids) is widely distributed in tropical and subtropical regions, accounting for approximately 80% of the sugar and 40% of the biofuel production worldwide. The cultivation of sugarcane relies on the vegetative propagation of stalks, leading to the accumulation of pathogenic microorganisms year-by-year, resulting in cultivar degeneration and quality degradation at both plant and ratoon crops [1,2]. The occurrence and prevalence of rust leads to reduced tillering numbers and thinner stems of sugarcane, giving rise to large-scale yield reductions (10–50%) and significant decreases in biomass (12–40%) [3,4]. Two types of rust have been found in sugarcane: brown rust caused by *Puccinia melanocephala* H. and *P. sydow*, and orange rust caused by *P. kuehnii* E. J. Butler [5]. Both rusts have been reported in the main sugarcane-growing countries, including Brazil, India, China, the United States, and Australia, which poses a great challenge to the safety and sustainable development of sugarcane cultivation [1,6].

In mainland China, brown and orange rust were reported in Yunnan and Fujian provinces in the early of 1980s, respectively, while the two sugarcane fungal diseases subsequently spread throughout China and have been major sugarcane diseases [7], resulting in 24.9% yield losses and a 3.1% sugar content reduction [8]. Currently, chemical fungicide application is an effective management strategy for controlling rust diseases [9]. In addition, the utilization of resistant cultivars developed through breeding programs is a longstanding and successful practice. Two methods of disease-resistance identification, natural inoculum pressure and artificial inoculation, are used for screening sugarcane resistance to rust in breeding programs [1]. The identification of resistance to brown rust in new elite sugarcane varieties under natural field conditions has been conducted in China, which indicated that 25% of varieties have high resistance to brown rust [10]. Notably, the main variety YZ08-1609 and some “ROC” serial varieties are highly resistant, but the other main varieties LC05-136, GT42, and GT44 are susceptible or moderately susceptible to brown rust [10]. This resistance to brown rust was identified by the artificial inoculation method in 31 wild core germplasms of the *Saccharum* complex, and seven tested clones of *Erianthus arundinaceus* were highly resistant to this disease [11]. Few reports were found about the identification of sugarcane resistance to orange rust in China.

The identification of sugarcane resistance to brown rust relies on molecular markers, of which *brown rust 1* (*Bru1*) is the most widely used. The self-bred progenies of the rust-resistant variety R570 showed an obvious separation ratio of 3:1 (resistant/susceptible) after inoculation with brown rust, and the resistance gene (*Bru1*) was consequently identified using amplified fragment length polymorphism (AFLP) and bulked segregant analysis (BSA) technologies [12]. A study by Costet et al. used 22 molecular markers linked to *Bru1* at an interval of 8.2 centimorgan (cM) in the variety R570 to analyze 380 sugarcane accessions, including modern cultivated varieties and breeding materials representing worldwide diversity. Their results revealed that 86% of 194 resistant sugarcane accessions carried the *Bru1* gene, indicating that this gene is a main source of resistance to brown rust in modern sugarcane varieties [13]. Subsequently, *Bru1* was widely used for sugarcane breeding for resistance to brown rust [14,15]. Regarding orange rust, some molecular markers, including the quantitative trait loci (QTLs), such as *G1* and *Oru*1/2/3/4/5, together with single nucleotide polymorphisms (SNPs) and insertion/deletions (InDels), have been linked to orange rust resistance in sugarcane, but the application of these markers is still limited in sugarcane breeding practices [16,17].

The *Saccharum* complex, including *Saccharum*, *Erianthus*, *Miscanthus*, *Narenga*, and *Sclerostachya*, shares common agronomic traits [18]. It is a crucial approach to introduce novel and elite resistance genes into sugarcane cultivars by distant hybridization between wild clones in the *Saccharum* complex and modern sugarcane varieties. Ensuring the resistance of wild germplasm resources is a priority procedure in sugarcane breeding programs. A study by Parco et al. showed that, among 1282 accessions from the world sugarcane germplasm resource database, the highest frequency of *Bru1* was found in *S. barberi* (79.3%) and *S. sinense* (71.8%), followed by *S. robustum* (59.1%), *S. officinarum* (26.4%), the interspecific hybrid species (21.0%), and *S. spontaneum* (18.8%) [19]. In China, the two markers R12H16 and 9O20-F4 were used to screen for *Bru1* in sugarcane clones and the results showed that 69.2% (18/26) of Chinese clones (resistant to brown rust) harbor this gene, which suggested that the donor of *Bru1* in these clones is possibly from *S. spontaneum* or *S. robustum* of New Guinea [14]. Unexpectedly, *Bru1* was absent in the clones of *E. arundinaceus* [11,14].

In this study, we investigated the distribution and incidence of the causal agents of rust in the main sugarcane planting provinces of China. Resistance identification for brown rust and molecular detection of *Bru1* markers among *E. arundinaceus* and their offspring were carried out. These findings will provide an important clue for preventing and controlling rust and supply some novel wild germplasm resources in sugarcane breeding for rust resistance.

## 2. Materials and Methods 

### 2.1. Collection of Rust Samples and Fungal Microscopy in China

A total of 201 leaf samples of *Saccharum* spp. hybrids with rust symptoms were collected from seven provinces of China, including Guangdong, Guangxi, Yunnan, Sichuan, Guizhou, Fujian, and Hainan, from 2013 to 2019. Another 88 clones consisting of *E. arundinaceus* offspring and their parents were from Hainan Breeding Research Base of Nanfan & Seed Industry, Guangdong Academy of Sciences (Guangzhou, China) during 2018–2019. After cleaning the leaf surface with 75% alcohol, the leaves were kept in a −80 °C refrigerator until further experiments. The information on these samples from *Saccharum* spp. hybrids is shown in Table S1. The samples of *E. arundinaceus* offspring and their pedigree information are listed in Tables S2 and S3.

Fungal spores were collected from rust-infected leaves of sugarcane for morphological analysis. The distilled water was dropped on a glass slide, and then spores were gently transferred into the water and spread evenly using a dissecting needle. The representative isolates of *P. kuehnii* and *P. melanocephala* were observed under a light microscope (BX53 microscope, Olympus, Hachioji, Japan).

### 2.2. Polymerase Chain Reaction (PCR) Detection of Rust

Two specific PCR primers Pm1-F (5′-AATTGTGGCTCGAACCATCTTC-3′)/Pm1-R (5′-TTGCTACTTTCCTTGATGCTC-3′) and Pk1-F (5′-AAGAGTGCACTTAATTGTGGCTC-3′)/Pk1-R (5′-CAGGTAACACCTTCCTTGATGTG-3′) were used to detect *P. melanocephala* and *P. kuehnii*, respectively [20]. Both primer pairs target the internal transcribed spacer (ITS1) region of fungal genomes of *P. melanocephala* and *P. kuehnii*. PCR amplification was performed using a VeritiPro thermal cycler (Thermo Fisher Scientific, Waltham, MA, USA). The total volume of 25 μL includes 1.0 μL DNA (about 100 ng) as the template, 1.0 U *Ex* Taq DNA polymerase, 2.5 μL 10 × *Ex* Taq buffer, 0.25 μM upstream and downstream primers, and 0.2 mM dNTPs. The reaction conditions were 94 °C for 5 min; 35 cycles of 94 °C for 30 s, 56 °C for 30 s, and 72 °C for 30 s; and 72 °C for 7 min. PCR amplification products were detected using 1.5% agarose gel electrophoresis.

### 2.3. Cloning and Sequencing of PCR Fragments

All PCR-positive products were purified and ligated into vector pMD19-T (TaKaRa, Dalian, China). Three positive clones per sample were picked up and sequenced with forward primer M13F (−47) and reverse primer M13F (−48). If the differences in nucleotide sequences at each site were simultaneously present two times in the same PCR amplifications, they were considered to be genetic variation. To determine the adaptive characteristics of *P. kuehnii* in China, the SNP analysis was performed on these PCR amplifications with PkPm-F/R primers in positive samples infected by *P. kuehnii*.

### 2.4. Multiple Sequence Alignment and Phylogenetic Analysis

A total of 62 sequences of *P. melanocephala* and 96 sequences of *P. kuehnii* were obtained from rust leaf samples with nucleotide lengths of 480 base pairs (bps) and 527 bps, respectively. In addition, some reference sequences of *P. melanocephala* (*n* = 11) and *P. kuehnii* (*n* = 39) from the United States, Argentina, Cuba, Australia, and Brazil were downloaded from GenBank (http://www.ncbi.nlm.nih.gov (accessed on 13 March 2024)). All sequence information is listed in Table S5. A phylogenetic tree was constructed using MEGA 11 software [21]. A phylogenetic tree was constructed using the neighbor-joining (NJ) method with default parameters in MEGA11. Robustness of the node of the phylogenetic tree was assessed from 1000 bootstrap replicates. Nucleotide sequence identity analysis was conducted using BioEdit 7.1.9 software [22].

### 2.5. Molecular Detection of Bru1 Gene

Four molecular markers, including R12H16-F/-R, 9O20-F4-F/-R, R12E03-1-F/-R, and R12E03-2-F/-R, were used to detect the *Bru1* gene (Table S4). The PCR volume and procedures were performed as described by Wang et al. [14]. The PCR amplification products of R12H16 were directly detected using 1.5% agarose gel electrophoresis. For 9O20-F4/*Hae*III, the PCR products were detected by *Hae*III restriction enzyme digestion (Thermo Fisher Scientific, Waltham, MA, USA), and then the band polymorphisms were analyzed. The *Bru1* gene was cloned and sequenced from sugarcane clones using four sets of molecular markers (R12H16, R12E03-1, 236 R12E03-2, and 9O20-F4). The SNP analysis was conducted on these *Bru1* sequences.

### 2.6. Collection of Spores of P. melanocephala

The spores of *P. melanocephala* were collected in Dehong, Yunnan Province (China) in October 2018 and 2019, following the protocol described by Li et al. [11]. Briefly, sugarcane leaves with brown rust symptoms were immersed in a bucket of water for 2 h, and then were gently rubbed by hand to collect spores. Solution containing spores in the bucket was filtered through two layers of gauze. The filtrated suspension was used for artificial inoculation. The number of spores was calculated using a blood cell-counting plate to obtain a final concentration of 10^4^ CFU/mL for inoculation.

### 2.7. Inoculation Method for Brown Rust

The artificial inoculation for brown rust was carried out in the greenhouse based on the protocol described by Li et al. [11]. Namely, the spore suspension was evenly sprayed on sugarcane leaves before the drops flowing on leaves. Subsequently, the plants were thoroughly covered by transparent plastic bags to increase air humidity. A total of 88 clones comprising 4 *E. arundinacus*, 71 *E. arundinaceus* hybrids, and 13 parental clones (12 *Saccharum* spp. and no *S. officinarum*) were inoculated (Table S2). The varieties R570 and Q124 acted as the controls for resistance and susceptibility to brown rust, respectively. The plants were grown in a greenhouse at 28 °C with a relative humidity of 80% in October 2018 and 2019.

### 2.8. Experimental Designs to Identify Resistance to Brown Rust

Single-bud stalk cuttings of offspring of *E. arundinaceus* and their parents were cleaned by hot water treatment at 50 °C for 2 h. Then, they were planted in 40 × 35 cm pots filled with 2/3 soil and organic matter (3:1.5) in each pot. The five-leaf-stage plants were used for artificial inoculation. The experiment was based on randomized block design each year using three biological replicates, each consisting of three pots with six plants per pot for each variety. A spore suspension of *P. melanocephala* was inoculated on plant leaves, while the monk-inoculation of sterile water was used as blank control. Incidence ratios and disease severities were observed and recorded five times every five days post inoculation. The classification standard of disease severity for brown rust is grades 1–9 [11]. Disease index (%) was calculated using the following formula [8]:

$$Disease\;index\left(\%\right)=\frac{\sum \left(the\;number\;of\;diseased\;plants\;at\;all\;grades\times corresponding\;grade\;value\right)}{highest\;grade\;value\times total\;number\;of\;plants}\times 100\%$$

PCR detection was conducted with Pm1-F/Pm1-R on some representative leaves to verify the *P. melanocephala* infection in each inoculated variety.

### 2.9. Data Analysis

A paired sample *t*-test was used to examine the statistically significant difference between disease indexes (the last time-point) among 88 clones of *E. arundinaceus* (HN92-77) hybrids and their parents together with 3 additional *E arundinaceus* clones between two years (2018 vs. 2019) (Table S3). A cluster analysis was performed with the Centetoid Method on disease indexes (the last time-point). All statistical analysis was performed using the SAS 9.2 software (https://support.sas.com (accessed on 20 January 2021)).

## 3. Results

### 3.1. Morphology of Rust Fungi in Sugarcane

**Figure 1:**
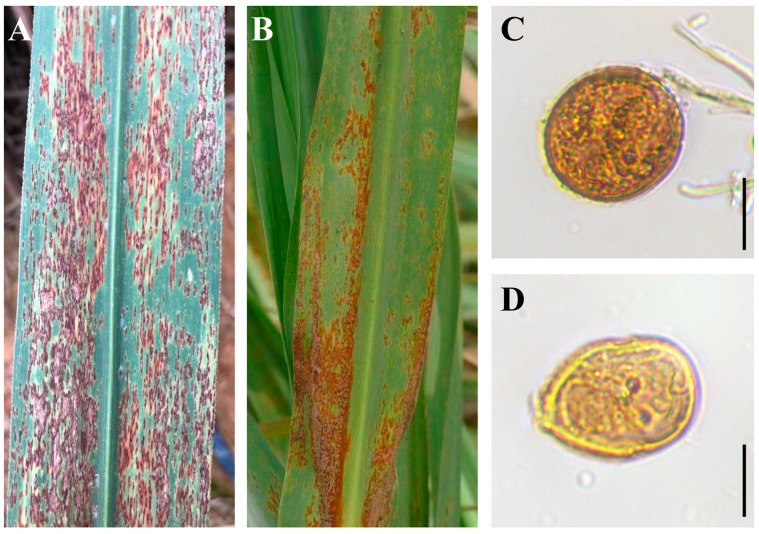
Disease symptoms and urediniospore morphologies of causal agents of brown rust and orange rust. (**A**,**B**) Symptomatic leaves collected from sugarcane cultivars YT03-373 and GT06-244, respectively. (**C**,**D**) Spore morphologies of *Puccinia melanocephala* and *P. kuehnii* as observed by microscopy, respectively. Scale bar = 20 µm.

To observe symptoms of rust and the morphology of casual fungi, representative leaf samples with rust symptoms were visually discriminated. The classical symptoms of brown rust were characterized by reddish-brown to brown lesions and sporulating pustules on the lower leaf surfaces (Figure 1A). In contrast, orange rust presented with orange to orange-brown lesions and pustules, lacking the darker brown pigmentation observed in brown rust. Notably, the pustules in orange rust formed clustered groupings on the lower leaf surfaces, distinguishing them from the more randomly distributed pustules associated with brown rust (Figure 1B). The morphologies of the urediniospores of brown rust were a dark color with no apical thickening (Figure 1C), while the urediniospores of orange rust were usually pear-shaped and orange to yellow-brown in color (Figure 1D).

### 3.2. Sugarcane Rust Detection Using PCR Method

To uncover the distribution and incidence of brown rust and orange rust in the main sugarcane planting provinces of China, a total 201 sugarcane samples was diagnosed by PCR with two primer pairs (Pm1-F/-R and Pk1-F/-R) for *P. melanocephala* and *P. kuehnii*, respectively (Figure S1). The sequencing results confirmed that these positive samples were indeed infected with one or both pathogenic fungi. Of the 201 samples, the infection rates of *P. melanocephala* and *P. kuehnii* were 17.9% and 34.8%, respectively, and the mixed infection rate was 12.9%. With the exception of samples from Fujian province, both *P. melanocephala* and *P. kuehnii* were detected in samples from all locations, including Guangdong, Guangxi, Yunnan, Hainan, Sichuan, and Guizhou province (Table 1).

### 3.3. Phylogenetic Analysis of P. melanocephala and P. kuehnii

**Figure 2:**
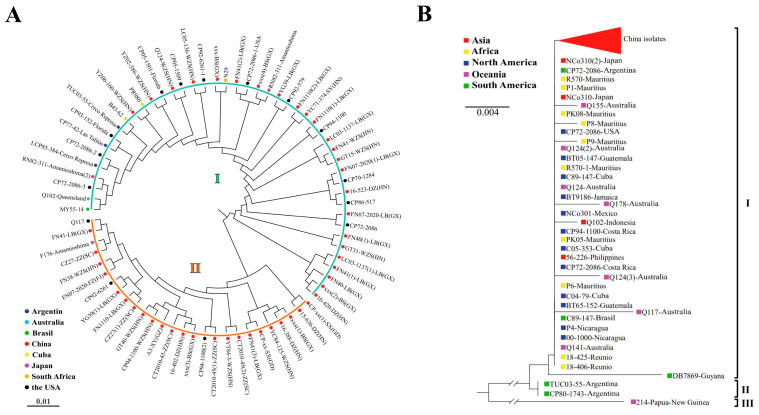
Phylogenetic analysis of *Puccinia melanocephala* (**A**) and *P. kuehnii* (**B**). Each phylogenetic tree was constructed using neighbor-joining (NJ) method with 1000 bootstrap replicates. Different colors of spherical and square shapes represent nucleotide sequences from different countries.

To understand the phylogenetic relationships of each causal fungus causing sugarcane rust, two phylogenetic trees were constructed based on the nucleotide sequences of ITS1 for *P. melanocephala* (this study = 62 and GenBank = 11) and *P. kuehnii* (this study = 96 and GenBank = 39). The phylogenetic analysis showed that the sequences of *P. melanocephala* could be divided into two groups (groups I and II) (Figure 2A), whereas the sequences of *P. kuehnii* could be clustered into three groups (I, II, and III) (Figure 2B). The sequences of *P. melanocephala* from China were distributed in both groups (Figure 2A) while the sequences of *P. kuehnii* from China were clustered in Group I (Figure 2B). The sequence identity analysis indicated that the Chinese isolates of *P. melanocephala* had 98.7~100.0% sequence identities and shared 94.7–100.0% identities with the foreign isolates (GenBank No. PP555955–PP556016). Meanwhile, the Chinese isolates of *P. kuehnii* had 97.6–100.0% sequence identities and shared 93.9–100.0% identities with the foreign isolates (GenBank No. PP556017–PP556112).

### 3.4. Single Nucleotide Polymorphism (SNP) Analysis of P. kuehnii

**Figure 3:**
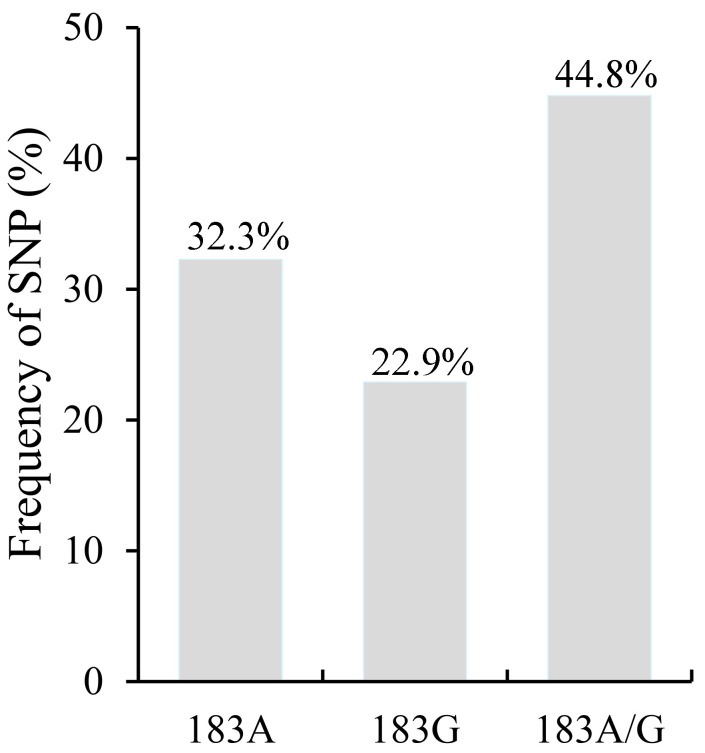
Frequency of single nucleotide polymorphisms (SNPs) on the 183rd site adenine/guanine (183A/G) in fragmental sequences by PCR amplification from positive samples infected by *P. kuehnii*.

To analyze the SNPs among *P. kuehnii* sequences, a total of 96 nucleotide sequences (606 bps) from PCR amplifications with the primers Pk1-F/Pk1-R were used for the SNP analysis. Among the 96 PCR fragments, 31, 22, and 43 sequences were adenine (183A), guanine (183G), or both bases (183A/G) at the 183rd nucleotide site, accounting for 32.3%, 22.9%, and 44.8%, respectively (Figure 3).

### 3.5. Detection of Bru1 Gene in E. arundinaceus Hybrids and Their Parents

**Figure 4:**
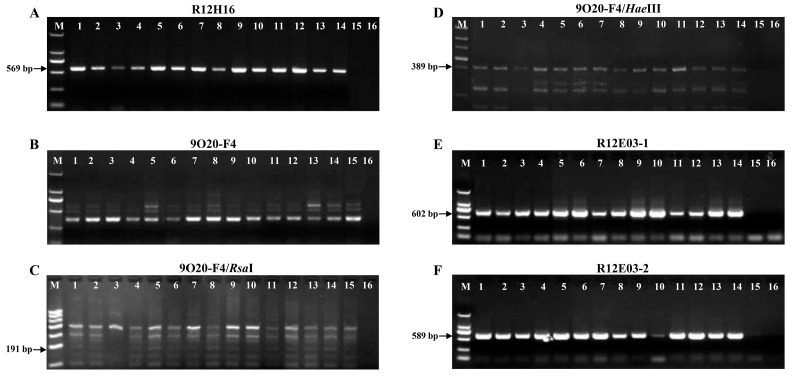
Occurrence of *Bru1* gene in 13 offspring of *Erianthus arundinaceus* detected by six molecular markers: R12H16 (**A**), 9O20-F4 (**B**), 9O20-F4/*Rsa*I (**C**), 9O20-F4/*Hae*III (**D**), R12E03-1 (**E**), and R12E03-2 (**F**). Arrows indicate target band of *Bru1.* M, DNA molecular weight marker; 1, resistant control R570; 2, YCE02-179; 3, YCE06-127; 4, YCE01-3; 5, YCE06-91; 6, YCE06-36; 7, YCE03-168; 8, YCE03-167; 9, YCE03-405; 10, YCE06-24; 11, YCE04-55; 12, YCE06-166; 13, YCE06-61; 14, YCE07-56; 15, susceptible control Q124; and 16, sterile H_2_O.

Four sets of molecular markers (R2H16, 9O20-F4, R12E03-1, and R12E03-2) were used for screening whether the *Bru1* gene was present in 88 *E. arundinaceus* hybrids and their parents. Of the 88 tested clones, 19 (21.6%) clones carrying *Bru1* were found, including 13 offspring of *E. arundinaceus* and 6 backcross parents of the “ROC” series (Table 2 and Figure 4). *Bru1* was also present in the resistant variety R570, but this gene was not detected in four *E. arundinaceus* varieties (Hainan92-79, Hainan92-91, Hainan92-77, and Hainan92-105), nor in *S. officinarum* (Badila) and their offspring (Table S2). *Bru1* is absent in the susceptible variety Q124. Notably, the clone YCE06-127 (no. 43 in Table 2) did not harbor the *Bru1* gene as revealed by 9O20-F4/*Rsa*I (191 bp target band was absent after enzyme digestion) (Figure 4C), whereas the opposite result was gained based on 9O20-F4/*Hae*III (389 bp target band was present after enzyme digestion) (Figure 4D).

### 3.6. SNP Analysis of Bru1 Gene

The SNP analysis was carried out on all sequences of the *Bru1* gene generated from four molecular markers (R12H16, R12E03-1, R12E03-2, and 9O20-F4) in the 19 above-mentioned clones carrying this gene (Figure S2). Among the *Bru1* sequences (570 bps) from the R12H16 marker, three clones (YCE06-24, YCE06-91, and YCE01-3) exhibited two SNP sites, 146T/C (thymine/cytosine) and 394C/G (cytosine/guanine). Among the *Bru1* sequences (600 bps) from the R12E03-1 marker, four clones (YCE04-55, YCE06-127, YCE06-24, and YCE01-3) had two SNP sites, 323C/A (cytosine/adenine) and 519T/C. Among the *Bru1* sequences (589 bps) from the R12E03-2 marker, three clones (YCE06-24, YCE01-3, and YCE06-127) exited three SNP sites, including 41C/A, 369A/G (adenine/guanine), and 543A/G, while the clone YCE04-55 was present in two SNP sites including 269A/G and 301T/C (thymine/cytosine). Among the *Bru1* sequences (200 bps) from the 9O20-F4/*Hae*III marker, four clones (YCE06-166, YCE06-24, YCE06-91, and YCE01-3) had three SNP sites, including 159T/C, 189A/G, and 230T/C. Notably, no SNP sites were found in seven clones (YCE02-179, YCE03-168, YCE03-167, YCE03-405, YCE06-61, YCE06-63, and YCE07-56) and six “ROC” clones (Figure S2).

### 3.7. Phylogenetic Analysis of Bru1 Gene

**Figure 5:**
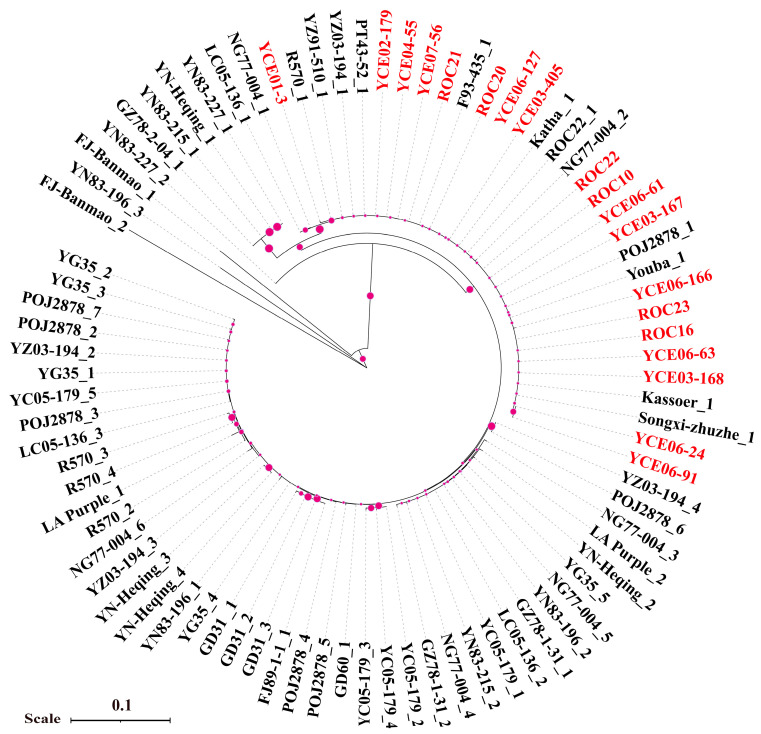
Phylogenetic tree of *Bru1* gene sequences generated from molecular marker 9O20-F4. Nucleotide sequences obtained in this study are represented by red letters. Other sequences with black color are from the published study. Purple circles indicate bootstrap values.

To explore the phylogenetic relationship of *Bru1* among 13 offspring of *E. arundinaceus* and 6 “ROC” varieties, a phylogenetic tree was constructed based on sequences generated from the molecular marker 9O20-F4 together with 65 according to sequences reported in the study by Wang et al. [14] (Figure 5). The 13 offspring of *E. arundinaceus* were clustered with clones of *Saccharum*, including NG77-004 (*S. robustum*), Youba_1 (*S. sinense*), Songxi-zhuzhe_1 (*S. sinense*), Katha_1 (*S. barberi*), R570 (cultivated species), and ROC22 (*Saccharum* spp.). The clone FJ-Banmao of *E. arundinaceus* was assigned into a unique evolutionary branch and the clone LA Purple of *S. officinarum* and the clones of *S. spontaneum* were clustered into a large evolutionary branch.

### 3.8. Identification of Resistance to Brown Rust in E. arundinaceus Hybrids and Their Parents

Resistance identification of brown rust was performed in 88 *E. arundinaceus* hybrids and their parents plus 2 control clones (R570 resistant and Q124 susceptible to brown rust) in October 2018 and 2019. The typical symptoms of brown rust were developed among clones after being inoculated by spores of *P. melanocephala* (Figure S3). These inoculated samples showing rust were controlled for the presence of *P. melanocphala* by PCR with primers Pm1-F/Pm1R. No significant difference was found in the disease indexes of these tested varieties between the two years (*p* = 0.992) (Table S2). Thus, the average disease indexes among 88 tested clones varied from 0 to 67.9%. Among them, 69.3% (61/88), 10.2% (9/88), and 9.1% (8/88) clones showed high resistance, resistance, and medium resistance to brown rust, respectively. Ten clones (11.4%) showed susceptibility to brown rust (Table S2).

**Figure 6:**
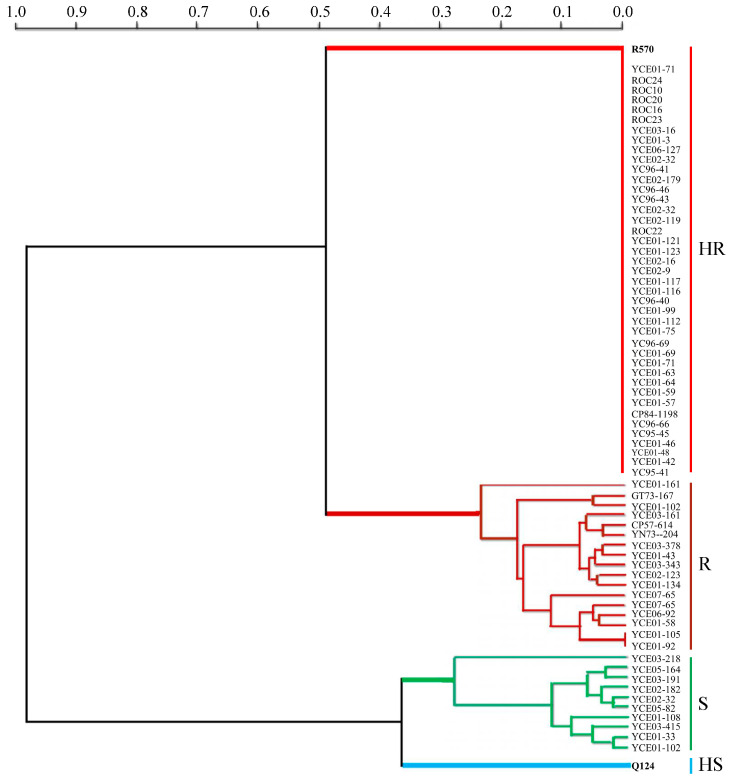
Clustering analysis of resistance to brown rust in offspring of *Erianthus arundinaceus* and some of their parents based on average disease indexes.

Based on the average disease indexes, resistance identification was conducted in the tested clones. Four categories were proposed (Table S2): (1) the control clone Q124 showed high susceptibility to brown rust (disease index = 67.9%); (2) 10 clones were susceptible to brown rust with disease indexes ranging from 30.6% (YCE03-218) to 36.4% (YCE01-102); (3) 16 clones were resistant to brown rust with disease indexes ranging from 5.8% (YCE01-92) to 22.9% (YCE01-61); and (4) the remaining 62 clones, including the resistant control clone R570, were highly resistant to brown rust (disease index = 0). The cluster analysis further supported the grouping by disease indexes (Figure 6).

## 4. Discussion

In China, the emergence of sugarcane rust occurred in the dominant cultivar F176 in Taiwan in the 1970s. The prevalence of sugarcane rust is strongly correlated with cultivar resistance, therefore making the development and deployment of resistant varieties the most economically effective strategy for controlling brown rust epidemics [7,10]. This study extensively assessed the prevalence of brown rust and orange rust in sugarcane in seven sugarcane planting provinces in China. Our results suggest that the two sugarcane rust diseases are widely disseminated in all surveyed provinces with single or mixed infections. Overall, orange rust caused by *P. kuehnii* is more prevalent than brown rust caused by *P. melanocephala* in China. *P. kuehnii* is a predominant fungus in the largest sugarcane planting area (Guangxi province), but *P. melanocephala* is a predominant pathogen in the second largest sugarcane planting area (Yunnan province). Similar results have been reported in previous studies [7,8]. Orange rust is an emerging major disease in some specific provinces, particularly in Guangxi, China.

The evolutionary arms race between plants and pathogens is a sophisticated process. Usually, novel virulent races that break through plant resistance genes often trigger rapid disease epidemics, therefore it is necessary to continuously monitor pathogenic variability in agricultural systems. Two races (1040 and 2143) of *P. kuehnii* occurred in Florida [5] and pathogenic variants of this pathogen (races PkSp01-01a4 and PkSp01-01) have also been identified in Brazil [23,24]. Four pathogenic races of *P. melanocephala* occurred in Florida [25], but there was no significant variation in the virulence of this causal agent in Brazil [26]. Notably, the variability in pathogenicity in *P. melanocephala* was associated with host genotypes [27]. Here, three phylogenetic groups of *P. kuehnii* and two groups of *P. melanocephala* were found in China. Although molecular evidence showed the occurrence of several SNPs in *P. kuehnii* and *P. melanocephala*, there is still a need for more in-depth investigations of the pathogenic variation in the two causal pathogens. Some SNPs present in *P. kuehnii* may be one of the main reasons for the prevalence of orange rust in various sugarcane areas in China [20]. Additionally, non-synonymous mutations or InDels in fungal genomic loci are possibly associated with the pathogenicity of pathogens, contributing to breakthroughs in the rust resistance of sugarcane breeding [20,28]. However, more biological experiments need to be explored in this inference.

Identification of resistance genes or loci is an effective and durable way to regenerate resistance varieties in sugarcane breeding programs. Some molecular markers, such as *Bru1* and *G1*, are closely linked to brown rust and orange rust-resistance genes in sugarcane, respectively [16,29]. The efficiency of the G1 marker for predicting rust resistance was higher than that of the *Bru1* marker [30]. Numerous investigations showed that the *Bru1* gene is extensively present in the *Saccharum* complex, including wild germplasms [11], modern sugarcane cultivars [10,13,14], and sugarcane accessions in Brazil [30] and worldwide [19]. In this study, the tested clones of *E. arundinaceus* were highly resistant to brown rust, but they did not contain the *Bru1* gene, which indicated that some alternative resistant genetic sites or genes possibly exist in these *E. arundinaceus* clones. Additionally, the interaction between sugarcane and races should be considered because the divergence in *P. melanocephala* pathogenicity was related to sugarcane genotypes [27].

It is noteworthy that some main sugarcane cultivars (LC05-136, GT42, and GT44) being planted in China are susceptible to sugarcane rust and they do not carry the *Bru1* gene [10]. Therefore, mining and the application of novel resistant gene sources from other wild germplasm resources in the *Saccharum* complex, such as *E. arundinaceus,* is a crucial strategy for improving sugarcane disease resistance in modern sugarcane breeding programs. In addition, there is an urgent need to develop more robust and accurate molecular markers for resistance identification in sugarcane rust, especially orange rust, which will speed up the sugarcane breeding process.

## 5. Conclusions

The application of resistant cultivars developed by breeding programs is an effective and efficient strategy for preventing and controlling sugarcane rust. In this study, artificial inoculation identification and molecular detection were conducted to determine the resistance to brown rust in *E. arundinaceus* hybrids and their parents. Most of the test clones showed resistance to brown rust, but only 24.4% of the resistant varieties harbored the *Bru1* gene. The *Bru1* gene in the offspring might be offered by “ROC” varieties. *E. arundinaceus* germplasm resources exhibit resistance to rust, but no *Bru1* gene was detected in these clones. These results suggest that other rust resistance genes might be present in the germplasms of *E. arundinaceus*. More in-depth investigations are needed to explore novel genes or genetic sites for rust resistance, which will help overcome the limitation of only relying on a single source of resistance. Notably, mining and the utilization of other disease-resistant genes from the *Saccharum* complex is an alternative strategy in genetic improvement programs of sugarcane.

## Figures and Tables

**Table 1:** Incidence rates (%) of sugarcane rust samples from seven main sugarcane planting provinces of China ^a^.

Province	No. of Tested Samples	Rates of *Pm*-Positive Samples	Rates of *Pk-*Positive Samples	Rates of Mixed Infection Samples	No. of Negative Samples
Guangxi	78	7.7% (6/78)	42.3% (33/78)	25.6% (20/78)	19
Yunnan	30	43.3% (13/30)	6.7% (2/30)	-	15
Guangdong	22	-	72.7% (16/22)	9.1% (2/22)	4
Hainan	45	33.3% (15/45)	22.2% (10/45)	6.7% (3/45)	17
Guizhou	12	8.3% (1/12)	58.3% (7/12)	-	4
Sichuan	9	-	22.2% (2/9)	11.1% (1/9)	6
Fujian	5	20% (1/5)	-	-	4
Total	201	17.9% (36/201)	34.8% (70/201)	12.9% (26/201)	69

^a^ *Pm*, *Puccinia melanocephala* causing brown rust; *Pk*, *P. kuehnii* causing orange rust.

**Table 2:** Molecular detection of *Bru1* in *E. arundinaceus* offspring and “ROC” varieties ^a^.

Sample No.	Clones	R12H16	9O20-F4	9O20-F4		R12E03-1	R12E03-2
				*Rsa*I	*Hae*III		
36	YCE02-179	+	+	+	+	+	+
42	YCE01-3	+	+	+	+	+	+
43	YCE06-127	+	+	−	+	+	+
44	YCE06-91	+	+	+	+	+	+
46	YCE06-63	+	+	+	+	+	+
48	YCE03-168	+	+	+	+	+	+
50	YCE03-167	+	+	+	+	+	+
54	YCE03-405	+	+	+	+	+	+
60	YCE06-24	+	+	+	+	+	+
61	YCE04-55	+	+	+	+	+	+
67	YCE06-166	+	+	+	+	+	+
68	YCE06-61	+	+	+	+	+	+
70	YCE07-56	+	+	+	+	+	+
78	ROC21	+	+	+	+	+	+
81	ROC16	+	+	+	+	+	+
82	ROC20	+	+	+	+	+	+
83	ROC10	+	+	+	+	+	+
84	ROC22	+	+	+	+	+	+
85	ROC23	+	+	+	+	+	+
RC	R570	+	+	+	+	+	+
SC	Q124	−	+	−	−	−	−

^a^ RC and SC represented resistant and susceptible controls, respectively; “+” and “−” indicated positive and negative results, respectively.

## Data Availability

The original contributions presented in this study are included in the article/Supplementary Material. Further inquiries can be directed to the corresponding authors.

## Supplementary Materials

The following supporting information can be downloaded at https://www.mdpi.com/article/10.3390/plants14081221/s1, Figure S1: PCR detection of *Puccinia melanocephala* (A) and *P. kuehnii* (B) from representative samples. Figure S2: Single nucleotide polymorphism (SNP) analysis of *Bru1* gene from 19 representative sugarcane clones. Figure S3: Brown rust symptoms present in clones/offspring from *Erianthus arundinaceus* post artificial inoculation by spores of *Puccinia melanocephala*. (A) YCE96-40 (F1); (B) YCE01-102 (BC1); (C) YCE03-415 (BC2); (D) HN92-77 (*E. arundinaceus*); (E) Q124 (susceptible control); and (F) R570 (resistant control); Table S1: Information about sugarcane leaves with rust symptoms sampled in fields. Table S2: Resistance grades of brown rust identified on 88 clones from *Erianthus arundinaceus* hybrids and their parents. Table S3: Pedigree information about *Erianthus arundinaceus* and their offspring. Table S4: Primer pairs used for detection of sugarcane rust diseases and molecular markers for *Bru1* gene in sugarcane. Table S5: Sequence information related to *Puccinia melanocephala* (480 bps) and *Puccinia kuehnii* (527 bps) used for phylogenic analysis.
