# Supplementary material for: Identification of Causal Agents of Rust of Saccharum spp. and Assessment of Resistance to Brown Rust in Erianthus arundinaceus Clones and Their Offspring
Source: Plants (Basel). 2025 Apr 16;14(8):1221. doi: 10.3390/plants14081221 (PMC12030035; doi:10.3390/plants14081221)
Supplement: Supplementary file 1 [file plants-14-01221-s001.zip › Supplemental Figures .pdf]

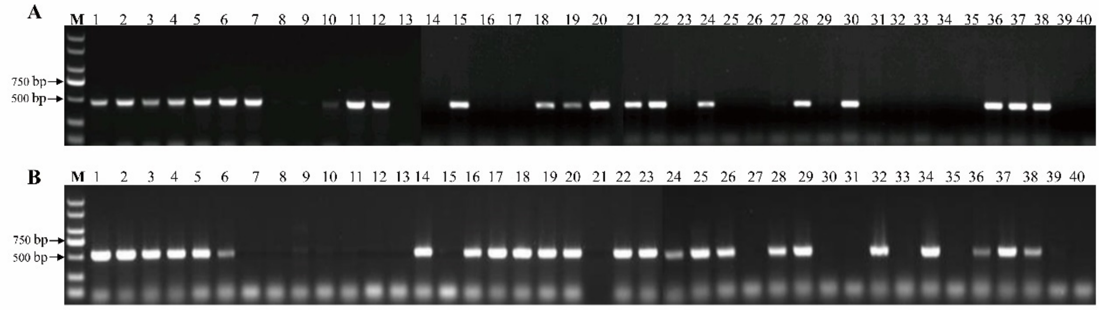

Figure S1. PCR detection of *Puccinia melanocephala* (A) and *P. kuehnii* (B) from representative samples.

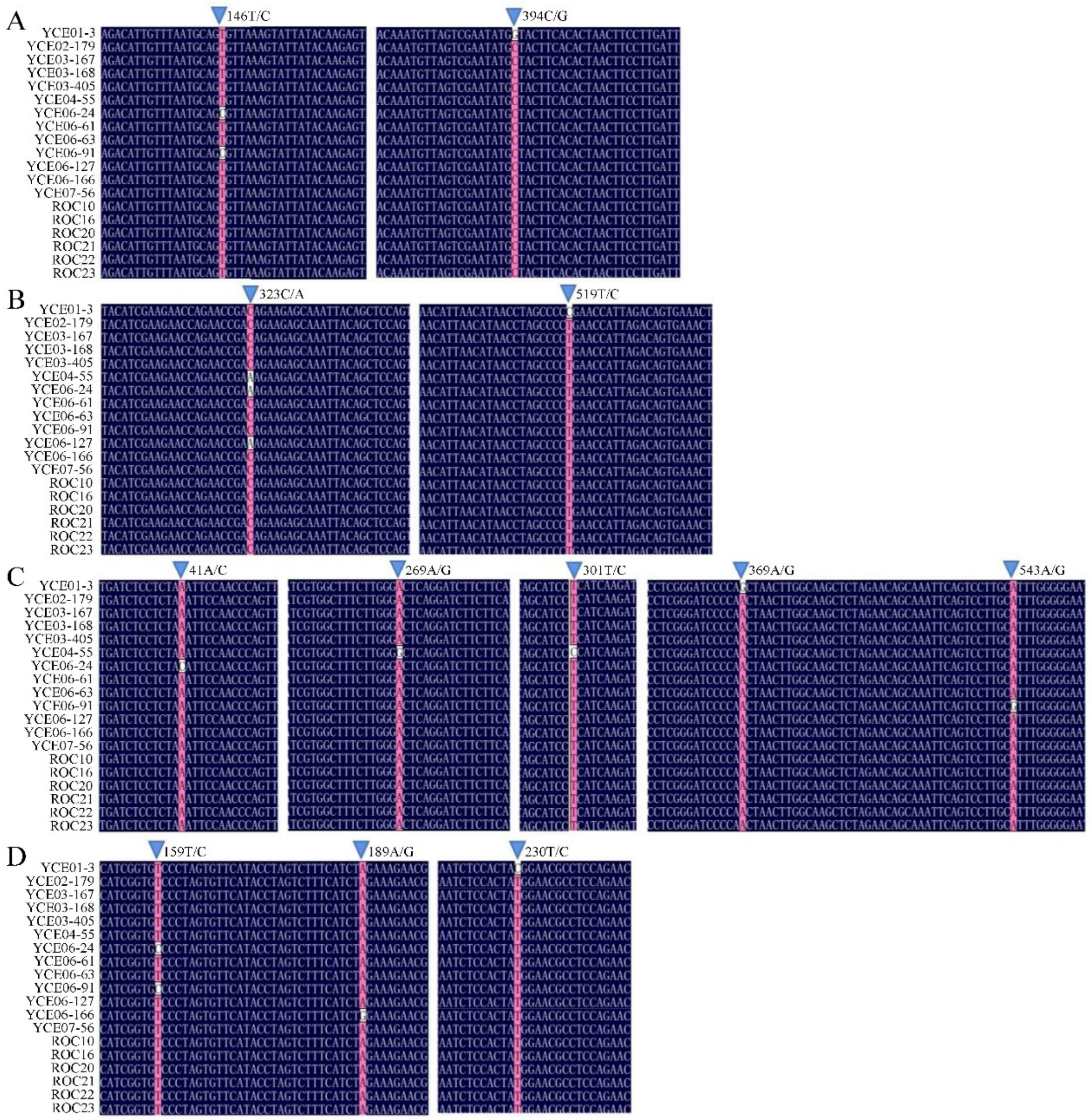

Figure S2. Single nucleotide polymorphism (SNP) analysis of *Bru1* gene from 19 representative sugarcane clones.

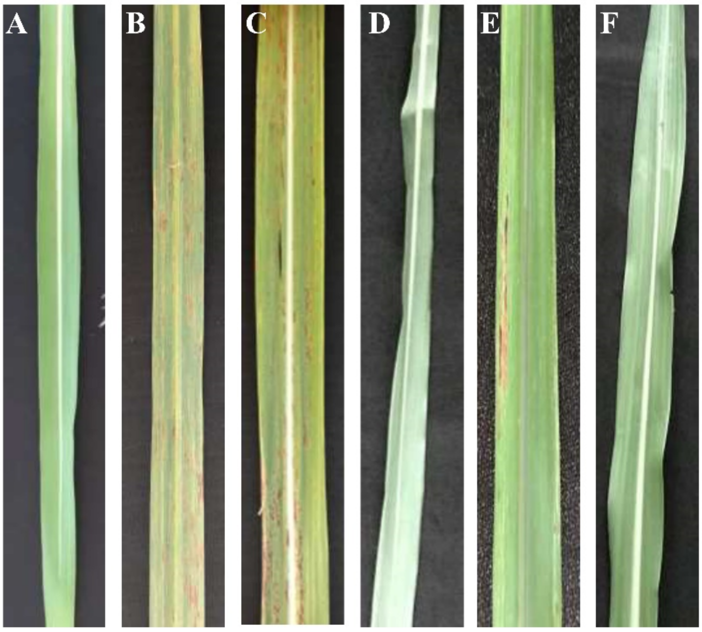

Figure S3. Brown rust symptoms present in clones/offspring from *Erianthus arundinaceus* post artificial inoculation by spores of *Puccinia melanocephala*. (A) YCE96-40 (F1); (B) YCE01-102 (BC1); (C) YCE03-415 (BC2); (D) HN92-77 (*E. arundinaceus*); (E) Q124 (susceptible control); and (F) R570 (resistant control).
